# Clinical supervision in public hospitals in Lesotho: Nurse managers’ support for registered nurses

**DOI:** 10.1371/journal.pone.0362115

**Published:** 2026-11-05

**Authors:** Mpho Zakaria Shelile, Molekodi Jacob Matsipane, Seepaneng Salaminah Moloko-Phiri, Miriam Mmamphamo Moagi

**Affiliations:** 1 Department of Nursing, Faculty of Health Sciences, National University of Lesotho, Maseru, Lesotho; 2 School of Nursing Science, Faculty of Health Sciences, North-West University, Potchefstroom, South Africa; 3 Nursing Department, Faculty of Health Sciences, University of Limpopo, Mankweng, South Africa; Khyber Medical University, PAKISTAN

## Abstract

**Background:**

Clinical supervision is regarded as essential for nurse managers in diverse healthcare settings to enhance nurse satisfaction, competence, and retention, leading to improved nursing care quality. The study aimed to explore and describe nurse managers’ support provided to registered nurses during clinical supervision at public hospitals in Lesotho.

**Methods:**

The study employed an exploratory descriptive qualitative research design to explore and describe the support provided to registered nurses during clinical supervision. Data was gathered through eight in-depth individual face-to-face interviews with participants purposefully selected from the public hospitals in Lesotho. Face-to-face interviews were recorded and transcribed verbatim for analysis. Data was analyzed by the researcher and the co-coder using thematic analysis as proposed by Braun and Clarke.

**Results:**

Three themes emerged from the data; namely: nurse managers’ supervisory dimensions to facilitate healthcare quality, nurse managers’ supervisory practices to capacitate and support registered nurses and nurse managers’ challenges with clinical supervision. The findings indicate that nurse managers provide structural support to registered nurses, apply various supervisory methods and processes, and supervise nursing practices in ways that relate to patient care outcomes during clinical supervision.

**Conclusion:**

Nurse managers guide registered nurses through performance monitoring, quality assurance, and resource supervision to uphold standards and policies. Supervision of registered nurses also support skill development, conflict management, and psycho-social well-being. However, challenges include addressing registered nurses’ attitudes, managing skill gaps, and overcoming nurse managers’ own knowledge limitations.

## Introduction

Clinical supervision is a structured, supportive professional development process in which registered nurses reflect on their practice within the context of confidentiality, safety, trust, and with a competent nurse supervisor [1]. Clinical supervision is a structured, collaborative, and developmental process aimed at enhancing professional competence, reflective practice, adherence to clinical standards, and patient safety. In contrast, managerial oversight primarily focuses on operational responsibilities and organisational performance [2].

Globally, clinical supervision is recognised as a critical component of nursing leadership that supports nurse managers in ensuring the delivery of safe and high-quality patient care across healthcare settings. Evidence indicates that structured clinical supervision strengthens professional accountability, promotes reflective practice, and contributes to improved patient care outcomes [1]. Traditionally, supervision in nursing was often characterised by hierarchical and authoritarian approaches that relied on directives and intimidation. However, such approaches have been shown to be ineffective in promoting professional development and collaborative learning, leading to a shift toward facilitative models of supervision that emphasise mentorship, support, and open communication between nurse managers and registered nurses [2]. Within these models, clinical supervision provides a structured opportunity for nurse managers and registered nurses to reflect on their practice in a supportive environment, thereby contributing to the improvement of clinical care standards.

The successful implementation of clinical supervision is influenced by several factors, including nurse managers’ knowledge, the frequency and duration of supervision sessions, and involvement in professional governance structures [3,4]. These elements contribute to structured and sustained supervision practices that promote professional accountability and continuous learning. Nurse managers therefore require adequate knowledge and skills to effectively supervise registered nurses and support the development of their professional competence [5]. In this regard, nurse managers play a key role in bridging the gap between clinical practice and professional development, ensuring that nursing care maintains high standards of safety and quality. An enabling organisational environment is also necessary to support effective clinical supervision. Adequate clinical supervision requires sufficient resources, appropriate supervisory skills, and clear procedures that guide supervision practices [6]. Without these enabling factors, supervision may become inconsistent and ineffective, limiting its potential to improve both nursing performance and patient outcomes.

Effective clinical supervisors therefore foster supervisory relationships built on mutual trust and respect [4]. In this role, nurse managers serve as professional role models who safeguard public safety and uphold professional standards by ensuring that supervisees meet required competency levels. Through these responsibilities, nurse managers not only support professional development but also reinforce accountability and ethical practice within healthcare settings. Similarly [7], recommend that clinical supervision in nursing should incorporate strategies such as observation, demonstration, feedback, and support in order to promote reflective practice and professional growth. The presence of structured guidelines and standards has also been emphasised as important for improving the acceptance and effectiveness of clinical supervision within healthcare organisations [8].

Through structured supervisory interactions, clinical supervision promotes alignment with organisational goals, professional standards, and continuing professional development while strengthening nurses’ clinical knowledge and decision-making skills [9]. Evidence also suggests that clinical supervision strengthens collaborative relationships between registered nurses, nurse managers, and other healthcare professionals, which ultimately improves patient care outcomes [10]. Effective supervision has been associated with reduced infection rates, decreased professional negligence, and increased feelings of professional recognition among nurses [11]. These outcomes demonstrate that clinical supervision not only improves patient safety but also enhances registered nurses’ morale and professional satisfaction.

Despite its benefits, there are notable challenges in applying clinical supervision. These challenges often arise from systemic constraints such as limited time, high workloads, and insufficient institutional support, which hinder consistent practice [12]. Leadership preparedness remains a significant challenge in many healthcare settings. Nurse managers often experience stress due to inadequate preparation for managerial roles, unclear job descriptions, and insufficient management training, all of which can negatively influence the effectiveness of clinical supervision [13]. Low engagement among staff and disruptions caused by heavy workloads have been identified as key barriers [9; 12]. These challenges can limit the consistency and effectiveness of clinical supervision, thereby reducing its potential benefits for both registered nurses and patients. Other challenges frequently arise from systemic constraints within healthcare systems, such as limited time, high workloads, and insufficient institutional support for supervision activities. Accessibility has also been identified as a concern, particularly for registered nurses working night shifts or during holidays, when nurse managers may not be available [14]. Such limitations create unequal opportunities for registered nurses to receive supervision, which may affect both professional development and the quality of care provided. In addition, effective supervision requires strong interpersonal skills. Supervisors must be able to create a safe and trusting environment that allows supervisees to openly discuss their clinical experiences and challenges [15]. Establishing such an environment encourages reflective practice and open communication, which are essential for learning and professional growth. Nevertheless, clinical supervision remains an important mechanism for professional reflection, as it provides registered nurses with opportunities to improve record keeping, build professional confidence, and enhance their overall well-being [16].

Despite growing recognition of its value, there remains limited empirical evidence regarding the optimal implementation of clinical supervision in nursing practice [17]. This gap has partly been attributed to the absence of standardised guidelines that clearly define how clinical supervision should be structured within healthcare systems. Similarly, there is limited evidence regarding the appropriate frequency and duration of clinical supervision sessions [18]. The lack of clarity regarding these aspects can hinder the consistent application of clinical supervision within public hospitals. Some scholars further argue that clinical supervision should focus on strengthening nursing processes and ensuring that nursing staff perform their duties efficiently and competently [19].

Research has shown that clinical supervision training programmes can significantly improve nurse managers’ supervisory skills, with positive effects sustained several months after the training [20,21]. Such programmes strengthen supervisory competence and support effective staff development. In addition, a supportive ward environment has been identified as an important factor in enhancing the effectiveness of clinical supervision. Supportive supervision can increase nurses’ confidence and reduce work-related stress [10], while structured guidelines help facilitate meaningful supervisory interactions [22]. However, the usefulness of certain supervision tools may vary. For example, some supervisors perceive checklists as cumbersome and redundant in routine clinical supervision [23]. This suggests that supervision tools must be practical and adaptable to specific clinical contexts. Resource constraints and psychological barriers also present challenges to effective supervision. Limited resources and inadequate mental preparedness among supervising nurses have been identified as key barriers to effective clinical supervision [24]. Similarly, nurses often expect stronger managerial support when dealing with complex clinical cases, highlighting the importance of accessible and competent supervision [25].

In Lesotho, these challenges may be intensified by broader socio-economic and structural pressures within healthcare systems. Lesotho’s health system faces ongoing challenges such as shortages of skilled healthcare personnel, high patient workloads, and limited opportunities for professional development. These conditions place considerable pressure on nurse managers who must simultaneously coordinate clinical services, manage staff shortages, and support the professional development of registered nurses. Within such environments, clinical supervision becomes an important mechanism for strengthening professional support and maintaining standards of nursing care.

However, in Lesotho clinical supervision remains largely unstructured and lacks a clear operational definition within clinical practice settings. Although initiatives such as the Nursing Education Partnership Initiative (NEPI) temporarily supported newly recruited nurses through mentorship and training, these interventions were not sustained and did not include experienced practitioners who may also require supervisory support [26]. In addition, the absence of a standardised supervision model and the lack of coordinated implementation across health facilities have contributed to inconsistencies in clinical supervision [27,28]. As a result, nurse managers often provide supervision informally without structured processes to guide supervisory interactions. Furthermore, despite the central role of nurse managers in clinical supervision, there is limited evidence on how this support is provided within the Lesotho context. Understanding nurse managers’ supervisory practices, the types of support they provide, and the challenges they encounter will address this knowledge gap and provide context-specific evidence to inform nursing management, policy development, leadership training, and interventions aimed at strengthening clinical supervision and ultimately improving patient care outcomes in public hospitals.

Given these contextual challenges, there is a need to better understand how nurse managers support registered nurses during clinical supervision within public hospital settings in Lesotho. Therefore, the study aimed to explore and describe nurse managers’ support provided to registered nurses during clinical supervision at public hospitals in Lesotho.

## Materials and methods

### Research design

This study employed a qualitative exploratory descriptive design to explore and describe the support provided by nurse managers to registered nurses during clinical supervision in public hospitals in Lesotho. This design was considered appropriate because it allowed the researcher to gain an in-depth understanding of the support provided by nurse managers to registered nurses during clinical supervision within their natural clinical settings. Qualitative design enabled the collection of rich, detailed data through direct engagement with nurse managers involved in clinical supervision and enabled participants to describe the support provided to registered nurses in their own words and allowed the researcher to capture the meanings they attributed to the support provided [29–31]. An exploratory descriptive design was suitable because there is limited evidence on nurse managers’ support during clinical supervision within the context of public hospitals in Lesotho. Where little is known about a phenomenon, exploratory descriptive research facilitates a comprehensive examination of participants’ experiences without imposing predetermined theoretical assumptions or variables [32]. The design allowed the researcher to obtain rich, detailed accounts of the support provided.

### Study setting

The study was conducted by the researcher at public hospitals in Lesotho, located in the districts of Mohale’s Hoek, Mafeteng, Maseru, Berea, Leribe, and Butha-Buthe. Eight hospitals were purposively selected for the study. These included one public hospital from Mohale’s Hoek district, one hospital from Mafeteng district, two public hospitals from Maseru district, two hospitals from Berea district, and one public hospital each from Leribe and Butha-Buthe districts. These hospitals also employ nurse managers who have formal responsibility for coordinating nursing services and providing clinical supervision to registered nurses, making them information-rich settings for addressing the study aim. Including hospitals from multiple districts increased the likelihood of obtaining a broad range of perspectives on how nurse managers provide support during clinical supervision while ensuring that the findings reflected variations in organisational environments, patient populations, and resource availability within the public health sector. Selecting public hospitals from several districts provided sufficient contextual diversity to generate a comprehensive understanding of the phenomenon under investigation.

### Population and sampling

The study population comprised nurse managers working in public hospitals in Lesotho. Eligible participants were those registered with the Lesotho Nursing Council and with a minimum of six months experience as a nurse manager in a public hospital. Non-probability purposive sampling was employed to select nurse managers who were able to actively contribute to the study [29]. This purposive sampling approach enabled the inclusion of participants who possessed essential knowledge relevant to the study [29]. Eight nurse managers participated in this study, with one participant recruited from each of the eight public hospitals included in the study. In the public hospital system in Lesotho, each hospital has one nurse manager who holds overall responsibility for nursing services, including planning, coordinating, monitoring, and supporting clinical supervision of registered nurses across the institution.

### Recruitment of the participants

An approval letter from the North West University Health Research Ethics Committee (NWUHREC) was submitted to the Ministry of Health in Lesotho to request permission to conduct the study in public hospitals. Subsequently, the researcher sent formal emails to the Director of Nursing Services (DNS) and the Head of Clinical Nursing Services (HCNS), acting as gatekeepers, to seek authorization to carry out the study in these hospitals. Once permission was granted on 28 April 2024, pamphlets were distributed to nurse managers inviting them to participate. The pamphlets included the contact details of the researcher, promoters, and the research assistant. All participants who took part in the study contacted the research assistant for further information, including scheduling appointments to provide written informed consent and participate in data collection. Participant recruitment was managed by the research assistant.

### Data collection

Data collection took place from 1 May 2024–31 July 2024. Data were collected through face-to-face interviews conducted at the participating public hospitals. Prior to data collection, the researcher explained the purpose of the study, the procedures involved, the potential risks and benefits, and participants’ rights, including their right to decline participation or withdraw from the study at any stage without penalty. Written informed consent was obtained from all participants before the interviews commenced. Participants were also asked for permission to audio-record the interviews, and recording only proceeded after consent had been granted. Confidentiality and anonymity were maintained throughout the study by assigning each participant a unique identification code and removing any personally identifying information from the interview transcripts and study reports.

To promote participants’ comfort, privacy, and confidentiality while minimising potential disruptions and social influences, interviews were held in a private and confidential room within each hospital that was mutually agreed upon by the researcher and the participant. The selected location ensured that conversations could take place without interruptions or being overheard, thereby facilitating open and honest discussion of participants’ support provided to registered nurses. The interview guide was specifically designed to address the study aim, with the central exploratory question being: “What support do you provide to registered nurses during clinical supervision?” Interviews were audio-recorded and lasted between 45 and 60 minutes. Open-ended questions were used to achieve the study goals, and field notes were maintained to capture all activities during the interviews, ensuring the accurate recording of essential information [29]. The researcher intended to conduct a minimum of twelve interviews, however, by the seventh interview, no new codes or themes emerged, and the information provided by participants became repetitive. An additional interview was conducted to confirm saturation. During the eighth interview, no new codes or substantive categories were identified, and participants consistently described similar supervisory support and challenges. Subsequent review of the coded data confirmed that additional interviews were unlikely to generate novel insights relevant to the research question. Saturation was therefore considered to have been achieved because the existing data were sufficiently rich and comprehensive to support well-developed categories and themes that adequately addressed the study aim.

### Data analysis

The researcher and an independent co-coder analyzed the data using the six steps of thematic analysis as outlined by Braun and Clarke [33]. The six-step thematic analysis approach proposed by Braun and Clarke [33] was selected because it provides a systematic, rigorous, and flexible method for identifying, analysing, and interpreting patterns of meaning within qualitative data. The approach was considered appropriate for this study as it sought to explore and describe nurse managers’ support provided to registered nurses during clinical supervision in public hospitals in Lesotho. Given the exploratory and descriptive nature of the study, thematic analysis enabled the researcher to examine participants’ practices without being constrained by a pre-existing theoretical framework. In the first step of data analysis, familiarization with the data, both the researcher and the co-coder read and re-read each verbatim interview transcript while comparing the transcripts with the audio recordings to ensure accuracy and immerse themselves in the data. Initial notes and observations were recorded to capture preliminary ideas and potential patterns relevant to the research question. The second step involved generating initial codes [33], the researcher and the co-coder independently conducted line-by-line coding of one transcript to identify meaningful units of data that reflected participants’ support for registered nurses. These meaningful units were assigned descriptive codes representing their underlying meaning. The researcher and the co-coder subsequently met to compare their independently generated codes, discuss similarities and differences, resolve discrepancies through consensus, and refine the coding framework by merging overlapping codes, clarifying code definitions, and removing redundant codes. The agreed coding framework was then applied systematically to the remaining transcripts, with new codes being created where data introduced concepts not captured by the existing framework. Similar codes that described related ideas were subsequently clustered together to form preliminary categories representing broader concepts within the dataset. During the third step, searching for themes, the researcher and the co-coder examined relationships among the categories to identify recurring patterns of meaning across participants’ accounts. Categories sharing similar underlying concepts were grouped together to develop candidate themes, while distinct but related aspects within each theme were organised into sub-themes. This iterative process involved moving back and forth between the coded extracts, categories, and the complete dataset to ensure that the emerging themes reflected participants’ support for registered nurses and addressed the research question.

The fourth step, reviewing themes, involved critically evaluating each candidate theme against the coded data and the entire dataset to determine whether the themes accurately represented the data and were internally coherent and distinct from one another. During this stage, some themes were merged, refined, separated, or discarded where appropriate, and categories and sub-themes were reorganised to improve conceptual clarity and ensure a logical relationship between the themes and the supporting data. The fifth step, defining and naming themes, the researcher and the co-coder refined the scope and content of each theme by identifying its central organising concept and clearly describing what distinguished it from the other themes. Sub-themes were reviewed to ensure that they meaningfully contributed to the overarching theme and accurately represented specific dimensions of participants’ account of support provided. Clear definitions and concise names were assigned to each theme and sub-theme to reflect their underlying meaning. The sixth and final step, writing up, the researcher and co-coder held several consensus meetings to verify the credibility and consistency of the final coding structure, categories, themes, and sub-themes before preparing the findings. Representative quotations from participants were selected to illustrate each theme and sub-theme and to demonstrate how the interpretations were grounded in the participants’ own words.

### Trustworthiness

The researcher ensured trustworthiness by following the four key principles: credibility, transferability, confirmability, and dependability [29].

### Credibility

Credibility was ensured through collection of field notes and observation of facial reactions collected through interviews. Reflexivity was maintained throughout the research process to acknowledge and minimise the potential influence of the researcher’s positionality on data generation, analysis, and interpretation. The principal researcher, being a nurse educator with experience in nursing practice and education, recognised that prior professional knowledge, assumptions, and expectations could shape interactions with participants, the framing of interview questions, and the interpretation of participants’ accounts. To manage this influence, the researcher engaged in continuous self-reflection before, during, and after each interview by documenting personal assumptions, emerging impressions, and methodological decisions in reflexive field notes. These reflections enabled the researcher to distinguish participants’ perspectives from preconceived ideas and to remain open to unexpected findings.

During data analysis, both researcher and the co-coder independently coded the data before comparing and discussing their interpretations to challenge individual assumptions and minimise subjective bias. Differences in coding and interpretation were resolved through discussion and consensus, ensuring that the themes were grounded in participants’ narratives rather than the researcher’s expectations. The researcher also repeatedly revisited the original transcripts to verify that the developing codes, categories, and themes accurately reflected the participants’ experiences. To further enhance credibility, methodological triangulation was employed by integrating data obtained from semi-structured interviews with field notes recorded during and immediately after each interview. Field notes provided contextual information on participants’ non-verbal communication, emotional expressions, and the interview setting, which complemented the interview transcripts and supported a richer interpretation of the findings. Maintaining an audit trail of methodological decisions, coding revisions, and theme development further enhanced the transparency and trustworthiness of the analytical process.

### Transferability

Transferability was enhanced by providing a thick description of the study context, participants, and research process. Detailed information was presented on the research setting, participants’ demographic and professional characteristics, and the contextual factors within which the study was conducted to enable readers to understand the circumstances under which the findings were generated. The research design, participant recruitment procedures, data collection methods, and data analysis process were described comprehensively to provide transparency regarding how the findings were produced. The sampling strategy, inclusion and exclusion criteria, and the rationale for selecting participants were explicitly described to demonstrate that participants were purposively chosen because they possessed rich knowledge and experience relevant to the research question. In addition, the characteristics of the participants and the context in which they worked were sufficiently detailed to allow readers to assess the similarity of their own settings to that of the study. Rather than claiming statistical generalisability, the provision of rich contextual and methodological detail enables readers to determine the relevance and potential transferability of the findings to comparable populations, healthcare settings, or contexts [29].

### Confirmability

Confirmability was enhanced by ensuring that the study findings were grounded in the participants’ accounts rather than the researcher’s personal assumptions or perspectives. Member checking was conducted by inviting participants to review and verify the accuracy of their interview transcripts and to clarify or expand on their responses where necessary, thereby ensuring that their views were accurately represented. Throughout the analytical process, reflexive notes and an audit trail documenting methodological decisions, coding revisions, category development, and theme refinement were maintained to provide transparency regarding how the findings were derived.

To minimise potential interpretive bias, the emerging codes, categories, themes, and their interpretations were regularly discussed with the co-coder and experienced qualitative researchers. These peer debriefing sessions provided opportunities to challenge assumptions, explore alternative interpretations, and ensure that the findings remained firmly supported by the data. Representative participant quotations were included in the presentation of the findings to demonstrate the link between the raw data and the researcher’s interpretations, thereby strengthening the confirmability of the study [29].

### Dependability

Dependability was enhanced by maintaining a transparent and well-documented research process that could be audited and replicated. An audit trail was established throughout the study, documenting methodological decisions, interview procedures, reflexive notes, coding decisions, category development, theme refinement, and changes made during data analysis. This documentation provided a clear record of how the findings evolved from the raw data.

To further strengthen dependability, an independent qualitative researcher with expertise in qualitative data analysis served as an auditor and co-coder. The independent coder was provided with the verbatim interview transcripts, field notes, the coding framework, and relevant methodological documentation to independently review and analyse the data. The researcher and independent coder subsequently compared their coding, categories, and emerging themes, discussing areas of agreement and disagreement until consensus was reached. Where discrepancies occurred, these were resolved through critical discussion and by revisiting the original transcripts to ensure that the interpretations accurately reflected participants’ accounts. This process enhanced the consistency, stability, and trustworthiness of the analytical process by demonstrating that the findings were supported by the data rather than by the researcher's individual interpretations alone.

### Ethical considerations

This study was approved by the NWU-Health Research Ethics Committee of the Faculty of Health Sciences of the North-West University (NWU-00175–23-A1) and the Ministry of Health of Lesotho (ID20–2024). Furthermore, the study adhered to the guidelines for research involving human participants as outlined in the 1969 Helsinki Declaration and the 2015 South African Department of Health’s *Ethics in Health Research* guidelines. The prospective participants were asked to read and sign the informed consent form if they were willing to participate in the study. Nurse Managers who needed more time to consider participation in the study were asked to submit the signed informed consent form in the provided sealed box at the mediator’s office at their convenience, agreeing that it would not take more than two (2) days. The independent person witnessed, signed, and collected the signed informed consent forms, then submitted them to the researcher in sealed envelopes. Before conducting the study, participants were fully informed about its purpose and the procedures involved. They were assured of their right to participate voluntarily, without any coercion, and were informed that they could withdraw from the study at any time. Participants were also made aware that no form of reward would be provided for their participation. Privacy was maintained by placing a “Do not disturb: session in progress” sign on the door, and anonymity was ensured by not recording participants’ names. The collected data were shared exclusively with the research team.

## Results

### Demographic data

The study included eight participants within a broad adult age range. The sample comprised participants of different sexes and educational backgrounds, including undergraduate and postgraduate nursing qualifications. Participants represented a range of nursing specialty areas. Their experience as nurse managers varied considerably, ranging from limited to extensive managerial experience. The participants represented diverse professional and educational backgrounds and met the established inclusion criteria.

### Themes and subthemes

Three main themes and eight sub-themes emerged from the qualitative analysis of individual face-to-face interviews’ data. The identified themes and sub-themes are presented in Table 1 below.

**Table 1:** Themes and Sub-themes.

Themes	Sub-themes
1. Nurse managers’ supervisory dimensions to facilitate healthcare quality	1.1Human resource support for registered nurses 1.2Material resource support for registered nurses 1.3Methods and processes to supervise registered nurses 1.4Supervision of registered nurses to enhance patient outcomes
2. Nurse managers’ supervisory practices to capacitate and support registered nurses	1.5Supportive supervision rendered to registered nurses 1.6Clinical supervision to capacitate registered nurses
3. Nurse managers’ challenges with clinical supervision	1.7Registered nurses related challenges 1.8Nurse manager related challenges

#### Theme 1: Nurse Managers’ supervisory dimensions to facilitate healthcare quality.

This theme describes how nurse managers support registered nurses through supervisory practices that contribute to quality healthcare delivery. Four subthemes were identified: human resource support, material resource support, supervisory methods and processes, and supervision aimed at improving patient outcomes.

Sub-theme 1.1: Human resource support for registered nurses: Participants described nurse managers as responsible for ensuring adequate staffing and effective workforce utilisation. This included advocating for recruitment, managing staff allocation across units, and approving duty schedules to maintain service continuity. One participant explained:

*“When I notice that we need more staff on the floor, especially when there are resignations, I must advocate for additional personnel… While the establishment list states that I should have 20 nurse midwives, I currently have 25. It’s my responsibility to advocate for these additional resources to ensure we meet our staffing needs effectively.”* ***(P8)***

One participant noted:

*“I manage the staff allocation across different units to optimise our workforce… unit leaders draw up the schedules, they share them with me for approval.”* ***(P7)***

Sub-theme 1.2: Material resource support for registered nurses: Participants reported that nurse managers monitor the availability and proper use of equipment and supplies necessary for nursing practice. Ensuring access to essential materials was considered important for enabling nurses to deliver effective care and preventing frustration caused by resource shortages.

One participant expressed:

*“It’s crucial to ensure that all necessary equipment is available, such as blood pressure machines, scales, and other essential tools.”* ***(P1)***

Another added:

*“We consistently ask registered nurses about their needs… Providing adequate resources, equipment, and supplies is crucial because when these items are lacking, it can lead to frustration among registered nurses and negatively impact the quality of care.”* ***(P4)***

Sub-theme 1.3: Methods and processes to supervise registered nurses: Participants described several supervisory mechanisms used to support registered nurses. These included providing access to clinical guidelines and policies, organising training sessions, conducting performance appraisals, and facilitating team meetings to address clinical challenges.

One participant explained:

*“I ensure that nurses have access to the latest clinical guidelines, protocols, and educational materials… Regular training sessions and workshops are organised to maintain access to the latest practices and standards.”* ***(P2)***

Another participant said:

*“I avail the clinical guidelines, educational material, and policies for them and take them through the policies as a way of supporting them in terms of ensuring that they perform according to the standards.”* ***(P7)***

Performance monitoring was also highlighted as part of supervision:

*“During performance appraisals, I ask questions about why certain actions were taken and what improvements are needed… when performance gaps are identified, I ask what kind of support they require.”* ***(P2)***

Interdepartmental meetings were also used to address operational challenges and improve coordination across departments.

Subtheme 1.4: Supervision of registered nurses to enhance patient outcomes: Participants indicated that clinical supervision contributes to quality assurance and patient care improvement through monitoring nursing practices, reviewing patient records, and engaging with nurses to address identified gaps.

One participant said:

*“After completing a ward round, I review the charts and engage with registered nurses to discuss my observations and any identified issues.”* **(P1)**

Another participant emphasised monitoring practices:

*“I conduct regular audits and monitoring of nursing practices to ensure adherence to established quality assurance standards.”* **(P2)**

Participants also highlighted the importance of patient feedback and conflict mediation in improving care outcomes.

#### Theme 2: Nurse Managers’ supervisory practices to equip and support registered nurses.

This theme describes how nurse managers provide professional and emotional support to registered nurses through supportive supervision and capacity-building initiatives.

Sub-theme 2.1 Supportive supervision rendered to registered nurses: Participants emphasised the importance of nurse managers being visible, approachable, and responsive to registered nurses’ needs. Supportive supervision included assisting with clinical tasks during high workloads, advocating for resources, and providing emotional support.

One participant said:

*“We have to be readily available for registered nurses when assistance is needed… nurse managers’ presence is especially crucial.”* ***(P1)***

Another participant explained:

*“When nurses reported being overwhelmed with patients, I would jump in to help… I ensure I’m present and responsive to their needs.”* **(P5)**

Participants also described advocacy as an important aspect of supportive supervision:

*“I believe one of the key supports I provide is advocating for my registered nurses… whether it's related to hiring or requests for equipment.”* **(P4)**

Emotional and psychological support for staff was also considered essential, particularly during challenging professional or personal situations:

*“I recognise the emotional demands of nursing and strive to create a supportive environment where nurses can share their challenges. I provide guidance and reassurance during difficult situations so they know they have someone to turn to.”* **(P2)**

Subtheme 2.2: Clinical supervision to capacitate registered nurses:

Participants reported that clinical supervision was also used to develop nurses’ clinical competencies. Nurse managers identified knowledge and skill gaps and addressed them through demonstrations, on-the-job training, and targeted development opportunities.

One participant noted:

*“When skill gaps or knowledge deficiencies in registered nurses are identified… tailored training and development opportunities are implemented.”* **(P2)**

Another participant added

*“I recognise that I possess more advanced knowledge and skills… This positions me to support them in addressing gaps in their clinical competencies.”* **(P3)**

#### Theme 3: Nurse Managers’ challenges with clinical supervision.

This theme highlights barriers experienced by nurse managers when implementing clinical supervision to support registered nurses. Two subthemes emerged: challenges related to registered nurses and challenges related to nurse managers themselves.

Sub-theme 3.1 Registered nurses’ related challenges: Participants reported challenges related to staff attitudes, accountability, and professional conduct. Some nurses were perceived as resistant to supervision or relied on legal documentation when disciplinary measures were taken.

A participant mentioned:

*“We face significant challenges… particularly in addressing attitudes among staff who frequently seek legal protection when reprimanded.”* **(P2)**

Another participant stated:

*“Many employees feel they need to operate under legal cover… even when we attempt to address violations of standards.”* **(P7)**

Participants also highlighted issues such as communication gaps, incomplete appraisals, and resistance to new systems.

Sub-theme 3.2: Nurse Manager-related challenges: Participants reported that nurse managers often assume supervisory roles without adequate preparation or ongoing leadership training. The absence of structured supervision frameworks and limited opportunities for professional development were identified as key barriers.

One participant explained:

*“It is essential to include nurse managers in capacity-building programs… without these opportunities, they may struggle to stay informed about the latest guidelines.”* **(P2)**

Another one added:

*“We need workshops or training focused on clinical supervision… the absence of such training leaves us at a disadvantage.”* **(P3)**

## Discussion

The primary aim of this study was to explore and describe the support provided to registered nurses by nurse managers during clinical supervision at public hospitals in Lesotho. The findings suggest that effective clinical supervision extends beyond monitoring nursing practice to ensuring that the human and material resources required for safe, high-quality care are available. Nurse managers’ active involvement in staff recruitment, allocation, and rotation demonstrates that clinical supervision is closely intertwined with workforce planning and resource management. Bohall and Bautista [34], describe clinical supervision as a mechanism to enhance nursing practice through staff management, and Achempim-Ansong et al. [11], highlight the centrality of human resource management in supervision. In the Lesotho context, inadequate staffing, such as expecting two registered nurses to care for 30 patients, compromises care quality and risks burnout, echoing challenges identified by Achempim-Ansong et al. [11]. Consistent with ElZeneny et al. [21], maintaining optimal nurse-to-patient ratios facilitate innovative supervisory practices and enhances training effectiveness. Budget constraints further complicated staffing decisions, a challenge supported by Ansah Ofei et al. [35], underscoring the need for strategic workforce planning in resource-limited settings. Zamanzadeh et al. [36] similarly link robust human resource support to improved patient outcomes.

Clinical supervision encompasses more than supporting nursing staff; it also requires nurse managers to ensure that the material resources necessary for safe and effective practice are available. Participants’ accounts suggest that the management of equipment availability, functionality, and accessibility is an integral component of supervisory practice rather than a separate administrative responsibility, consistent with findings by Buus and Gonge [37] and Saab et al. [9]. Ensuring the presence of essential tools, from blood pressure machines to patient charts, aligns with Akpor et al. [38], who note that well-maintained equipment enhances care quality, and Kucera et al. [39], who link inadequate resources to job dissatisfaction and compromised care. In Lesotho’s public hospitals, nurse managers engaged in procurement and workflow optimisation, resonating with Blackman et al. [40], who emphasise the relationship between structured ward layouts, supply availability, and nursing productivity.

Structured supervisory methods are essential mechanisms through which nurse managers promote accountability, consistency, and quality in clinical practice. The use of written policies, workshops, performance appraisals, and interdepartmental meetings reflects a deliberate and systematic approach to clinical supervision that extends beyond routine oversight. These practices align with O’Keeffe and James [41] and Fernandez et al. [42], who underscore the role of supervision in enforcing protocols and promoting patient safety, and with ElZeneny et al. [21], who highlight the importance of clearly defined roles in clinical oversight. Monitoring and evaluation through goal setting, feedback, and performance appraisals enhance accountability, consistent with Snowdon et al. [5] and Achempim-Ansong et al. [11]. Regular interdepartmental meetings further facilitated collaborative problem-solving and communication, corroborated by Efendy and Prisantika [43], Raliphaswa et al. [24], and Holst and Severinsson [44], emphasizing that structured forums improve care outcomes in complex hospital environments.

Clinical supervision serves as an important quality assurance mechanism through which nurse managers safeguard patient outcomes. Participants’ descriptions of conducting clinical audits, reviewing patient records, and directly observing nursing practice indicate that supervision extends beyond monitoring staff performance to systematically evaluating the quality and safety of patient care. This is consistent with Mohamed and Ahmed [45], who advocate for evidence-based supervisory evaluation, and Severinsson [46] and Yuswanto et al. [16], who highlight continuous quality assessment as essential for effective supervision. Participants reported using real-time supervision and teaching moments to correct deficiencies, reinforcing Magge et al. [47] and Di Maio et al. [48], who link clinical supervision to process improvement and reduced infection rates. Prioritising individualised care and patient feedback mirrors Martin et al. [49], demonstrating that attentive clinical supervision enhances satisfaction and clinical outcomes. Effective clinical supervision enhances patient outcomes, ensures accountability, and improves the quality of nursing care. Through performance monitoring, quality assurance, and supervision of human and material resources, nurse managers guide registered nurses to adhere to nursing standards, policies, and procedures.

Supportive supervision is a relational and developmental process that extends beyond managerial oversight to fostering professional growth and confidence among registered nurses. Nurse managers’ physical presence in the clinical area, provision of hands-on guidance, and ongoing mentoring create opportunities for real-time learning, problem-solving, and emotional support. This reflects relational and coaching leadership paradigms, as noted by Johansson et al. [50] and Bellerose et al. [51], who highlight the importance of accessibility and engagement in fostering effective clinical supervision. Achempim-Ansong et al. [11] further reinforce that time spent in clinical areas allows managers to provide immediate guidance, particularly in high-demand contexts such as maternity or antiretroviral treatment wards. Nurse managers also served as mediators in conflict resolution, advocating for fair and neutral approaches to disputes, consistent with Yuswanto et al. [16] and Love et al. [6], thereby promoting a cohesive and supportive work environment.

Emotional and psychological supports are core supervisory function, extending to staff well-being and work-life challenges. Nurse managers provide counselling, wellness interventions, and proactive engagement with registered nurses’ families, corroborating findings by Circenis et al. [52], Efendy and Prisantika [43], O’Donnell et al. [53], and Rothwell et al. [18], all of whom link supervision to reduced burnout, stress alleviation, and enhanced job satisfaction. Advocacy for staffing, resources, and recognition further reinforced registered nurses’ professional value, aligning with Love et al. [6].

Capacity-building through clinical supervision is a critical mechanism for enhancing nursing competence. Participants described hands-on demonstrations, tailored training, and peer-learning initiatives to bridge skill gaps, echoing King et al. [54], Jorgensen et al. [55], Cook et al. [56], and Abreu and Marrow [57]. Continuous professional development, facilitated through structured supervision, reinforced both individual skills and team-wide commitment to excellence, in line with Achempim-Ansong et al. [11]. Clinical supervision is essential for knowledge and skill development and ensuring that registered nurses have competencies to meet the demands of patient care [58]. It focuses on creating an environment where registered nurses feel their nurse managers are accessible, approachable, and responsive to their needs, while also effectively managing conflicts and providing psychosocial support.

Despite these strengths, nurse managers face challenges, including managing staff attitudes, negligence, legalistic behaviour, and union interventions, which complicate accountability and clinical supervision [6,24,59]. Supervisory efficacy is hindered by gaps in nurses’ knowledge, self-confidence, and professional skills [6,55,60]. Furthermore, the lack of standardised frameworks and structured supervisory tools limited consistency in clinical supervision, as documented by Driscoll et al. [61], Snowdon et al. [5], O’Donnell et al. [53], Noor et al. [20], Yuswanto [16], Saab et al. [9], Nugrahini and Pohan [62], and Aparício and Nicholson [63]. These findings indicate that targeted capacity-building, structured supervision models, and ongoing professional development are essential for effective nurse management within Lesotho’s public hospital context, ensuring both staff competence and patient safety. Nurse Managers encounter several challenges during clinical supervision, including addressing issues related to registered nurses’ attitudes to ensure they align with organisational values, managing nurses who may lack essential knowledge and skills, and dealing with their limitations in knowledge and capacity to effectively perform clinical supervision.

## Conclusion

This study shows the central role of nurse managers in enhancing the quality of nursing care through comprehensive clinical supervision within Lesotho’s public hospitals. Their supervisory role encompasses strategic human and material resource management, structured supervisory processes, supportive mentorship, and capacity-building initiatives that directly influence patient outcomes. The findings indicate that effective clinical supervision relies on both the accessibility and professional competence of nurse managers, as well as their ability to navigate institutional constraints such as staffing shortages, budget limitations, and lack of standardised frameworks. Moreover, nurse managers’ advocacy, conflict resolution, and emotional support for registered nurses reinforce a positive work environment and ensure professional accountability. However, challenges such as knowledge gaps among nurse managers and registered nurses, resistance to supervision, and inconsistent supervisory structures reflect the need for targeted capacity-building, structured supervision frameworks, and ongoing professional development. Collectively, these insights advance understanding of how nurse managers operationalise clinical supervision in resource-constrained public healthcare settings, providing evidence to inform policy, training, and organisational strategies aimed at strengthening nursing leadership and improving patient care.

### Limitations of the study

Researcher reflexivity may have influenced data generation and interpretation, as the researcher’s professional background in nursing and familiarity with the clinical context could have shaped interactions with participants and the interpretation of findings, although reflexive awareness and systematic analytic procedures were applied to minimise bias. While efforts were made to achieve data saturation, the study involved a relatively small sample of nurse managers from public hospitals, which may not fully represent the support provided to registered nurses during clinical supervision across other healthcare settings in Lesotho. Recruitment constraints may also have affected the depth and diversity of the data, as participation depended on nurse managers who were available and willing to participate.

### Contribution to new knowledge

This study contributes new knowledge by providing context-specific insight into how nurse managers in public hospitals in Lesotho operationalise clinical supervision within resource-constrained healthcare settings. While previous studies have largely examined clinical supervision as a structured professional development mechanism, the findings of this study demonstrate that, in the Lesotho context, clinical supervision extends beyond formal oversight to include workforce coordination, resource advocacy, and psychosocial support for registered nurses. The study further shows how nurse managers integrate supervisory practices with broader managerial responsibilities, such as staffing allocation, interdepartmental coordination, and quality assurance processes, in order to sustain nursing care delivery despite systemic constraints. The findings further reveal context-specific challenges, including legalistic workplace dynamics and limited leadership preparation for nurse managers, which are less frequently documented in the clinical supervision literature. By situating clinical supervision within the realities of a resource-limited health system, this study advances understanding of the multifaceted supervisory roles of nurse managers and provides empirical evidence that can inform leadership support strategies in similar low-resource healthcare contexts.

## Supporting information

S1 Table Themes and Subthemes. The data used in this study can be downloaded from the following link: https://doi.org/10.6084/m9.figshare.33098735.
(DOCX)
