# Supplementary material for: Clinical supervision in public hospitals in Lesotho: Nurse managers’ support for registered nurses
Source: PLoS One. 2026 Nov 5;21(11):e0362115. doi: 10.1371/journal.pone.0362115 (PMC13739098; doi:10.1371/journal.pone.0362115)
Supplement: Supplementary file 1 [file pone.0362115.s001.docx]

*Table 1: Themes and Sub-themes*

| **Themes** | **Sub-themes** |
| --- | --- |
| 1. Nurse managers' supervisory dimensions to facilitate healthcare quality | 1.1. Human resource support for registered nurses 1.2. Material resource support for registered nurses 1.3. Methods and processes to supervise registered nurses 1.4. Supervision of registered nurses to enhance patient outcomes |
| 2. Nurse managers' supervisory practices to capacitate and support registered nurses | 2.1. Supportive supervision rendered to registered nurses 2.2. Clinical supervision to capacitate registered nurses |
| 3. Nurse managers' challenges with clinical supervision | 3.1. Registered nurses related challenges 3.2. Nurse manager related challenges |
